# NZ-GMP Approved Serum Improve hDPSC Osteogenic Commitment and Increase Angiogenic Factor Expression

**DOI:** 10.3389/fphys.2016.00354

**Published:** 2016-08-19

**Authors:** Anna Spina, Roberta Montella, Davide Liccardo, Alfredo De Rosa, Luigi Laino, Thimios A. Mitsiadis, Marcella La Noce

**Affiliations:** ^1^Sezione di Biotecnologie, Dipartimento di Medicina Sperimentale, Istologia Medica e Biologia Molecolare, Seconda Università degli Studi di NapoliNapoli, Italy; ^2^Sezione di Odontostomatologia, Dipartimento Multidisciplinare Medico-chirurgico, Seconda Università degli Studi di NapoliNapoli, Italy; ^3^Dipartimento di Medicina Clinica e Sperimentale, Università degli Studi di FoggiaFoggia, Italy; ^4^Orofacial Development and Regeneration, ZZM, Institute of Oral Biology, University of ZurichZurich, Switzerland

**Keywords:** hDPSCs, osteogenesis, angiogenesis, bone differentiation, bioscaffold

## Abstract

Human dental pulp stem cells (hDPSCs), selected from the stromal-vascular fraction of dental pulp, are ecto-mesenchymal stem cells deriving from neural crests, successfully used in human bone tissue engineering. For their use in human therapy GMP procedures are required. For instance, the use of fetal bovine serum (FBS) is strongly discouraged in clinical practice due to its high risk of prions and other infections for human health. Alternatively, clinical grade sera have been suggested, including the New Zealand FBS (NZ-FBS). Therefore, the aim of this study was to evaluate the behavior of hDPSCs expanded in culture medium containing NZ-FBS. Since it was widely demonstrated hDPSCs display relevant capabilities to differentiate into osteogenic and angiogenic lineages, we performed a comparative study to assess if these features are also retained by cultivating the cells with a safer serum never tested on this cell line. hDPSCs were grown using NZ-FBS and conventional (C-FBS) for 7, 14, and 21 days, in both 2D and 3D cultures. Growth curves, expression of bone-related markers, calcification and angiogenesis were evaluated. NZ-FBS induced significant cell growth with respect to C-FBS and promoted an earlier increase expression of osteogenic markers, in particular of those involved in the formation of mineralized matrix (BSP and OPN) within 14 days. In addition, hDPSCs cultured in presence of NZ-FBS were found to produce higher mRNA levels of the angiogenic factors, such as VEGF and PDGFA. Taken together, our results highlight that hDPSCs proliferate, enhance their osteogenic commitment and increase angiogenic factors in NZ-FBS containing medium. These features have also been found when hDPSC were seeded on the clinical-grade collagen I scaffold (Bio-Gide®), leading to the conclusion that for human therapy some procedures and above all the use of GMP-approved materials have no negative impact.

## Introduction

There is an increasing interest in the regeneration of organs and tissues damaged by diseases, trauma or aging. In human regeneration, the use of autologous and allogeneic stem cells isolated from various tissues of adult individuals has fewer ethical problems than the use of embryonic stem cells (Caplan, 2007; Ferro et al., 2012). An easily accessible source of mesenchymal stem cells is the dental pulp, which is a stromal, fibrous, highly vascularized structure located in the inner part of the tooth (d'Aquino et al., 2007). Stem cells from dental follicles and human dental pulp stem cells (hDPSCs) express several transcription factors that are involved in the maintenance of self-renewal and pluripotency such as Sox2 and Nanog (d'Aquino et al., 2011), and specific mesenchymal stem cell markers such as CD90 (Ponnaiyan et al., 2012) and CD34, used for the first time by Laino et al. to isolate a population of stromal stem cells of neural crest origin (Laino et al., 2005). Under definite culture conditions, hDPSCs differentiate into numerous cell types, including osteogenic, adipogenic, neurogenic, and neural crest-derived cells (Almushayt et al., 2006; Zhang et al., 2006; Arthur et al., 2008; Paino et al., 2010). Several studies have shown that hDPSCs can be successfully used in clinical practice as a new therapeutic tool for healing bone defects (d'Aquino et al., 2009; Mitsiadis et al., 2011; Giuliani et al., 2013) since it was demonstrate that DPSCs are self-committed to osteogenic and angiogenic differentiation (d'Aquino et al., 2007).

However, various important issues have been addressed concerning the clinical use of hDPSCs, namely the generation of an adequate number of cells in a short period of time; the characterization of cells at all stages of culture before their use in clinic (Tirino et al., 2012); and the development of safe-for-human-use materials for the isolation and maintenance of cells selected for therapy (Desiderio et al., 2014). For these reasons, it is necessary to standardize procedures and good manufacturing practice (GMP) protocols for the culture of the stem cells for clinical use (La Noce et al., 2014). To this end, the development of international standards for the production of hDPSCs, in accordance with GMP, holds a paramount importance.

In this context, physical and chemical factors play a pivotal role since they can influence the cell culture conditions and, therefore, the cells' fate. Stem cells display different behaviors and answers in culture and it is extremely complex to obtain similar stem cell stocks following a standard protocol. Moreover, the serum added to basic medium may contain growth factors and nutrients that mimic the extracellular environment, which may be crucial for cell proliferation and differentiation. Usually, cells are cultured in media containing animal-origin sera which are not recommended for clinical practice because of concerns about human safety. In fact, viruses, prions, mycoplasma or any other animal pathogen could contaminate the sera. Bovine sera are the most commonly used and, in particular, fetal bovine serum (C-FBS) has become the standard supplement for cell culture media. It is a cocktail of most of the factors required for cell proliferation and maintenance, and thus is an almost universal growth supplement. FBS sourced from New Zealand (NZ-FBS) is a valid bovine serum as it offers greater safety due to the fact that New Zealand has the fewest reported bovine diseases in the world for its geographically isolation. Indeed, one of the benefits of this isolation is that New Zealand has been determined to be free from BSE and FMD.

Additionally, the use of DPSCs in clinical applications requires a support system. The choice of scaffolds for tissue engineering is rather critical due to the large amount of existing variables and the GMP procedures require that these scaffolds are clinical-grade, ready for human use (Naddeo et al., 2015; Mele et al., 2016).

In this study, two different sera have been assayed and compared, namely the commonly used C-FBS, and a more controlled FBS, originating from New Zealand (NZ-FBS), that is GMP-approved. In addition, the influence of two different sera on a three-dimensional (3D) culture of hDPSC *in vitro*, using a commercially available clinical-grade collagen I bioscaffold, the Bio-Gide® (Geistlich, Wolhusen, CH), has been assessed.

## Materials and methods

### Human dental pulp extraction and cell culture

Human dental pulps were extracted from teeth of healthy adults (21–38 years of age). Prior to the extraction, each subject (*n* = 40) was checked for systemic and oral infections or diseases. Only patients undergoing a third molar or supernumerary tooth extraction were interviewed and enlisted. All subjects signed the Ethical Committee (Second University Internal Ethical Committee) consent brochure before being enrolled. Every subject was pretreated for a week with professional dental hygiene. The dental crown was covered with 0.3% chlorhexidine gel (Forhans, New York, USA) for 2 min prior to the extraction. Dental pulp was obtained with a dentinal excavator or a Gracey curette. The pulp was delicately removed and immersed for 1 h at 37°C in a digestive solution composed of 3 mg/ml type I collagenase and 4 mg/ml dispase in phosphate buffered saline (PBS) containing 40 mg/ml gentamicin. Once digested, the solution was filtered through 70 μm Falcon strainers (Becton & Dickinson, Franklin Lakes, NJ, USA). Cells were cultured in basal growth medium consisting of Dulbecco's modified Eagle's medium (DMEM) with 100 U/mL penicillin, 100 mg/mL streptomycin and 200 mM L-glutamine (all from GIBCO, Monza, Italy), supplemented with the two sera to get the different culture conditions: (a) 10% fetal bovine serum (C-FBS; GIBCO, Monza, Italy), and (b) 10% New Zealand origin FBS (NZ-FBS; SAFC Biosciences). Cultures were maintained in a humidified atmosphere under 5% CO_2_ at 37°C. Media were changed twice a week. The analyses were conducted at 7, 14, and 21 days of culture.

### Growth analysis

Cells in the two different culture media were plated at a density of 50,000 cells/well in 6-well plates and at a density of 100,000 cells in T25 flasks. The cells were harvested and re-suspended in PBS. An aliquot of cell suspension was diluted with 0.4% trypan blue (Sigma-Aldrich, Milan, Italy), pipetted onto a hemocytometer and counted under a microscope. The number of viable cells for each experimental condition was counted and represented on a linear graph. The doubling time (DT) was determined from the growth curves or by using the formula.

$$\begin{array}{l} \text{DT}=\left(\text{t}-{\text{t}}_{0}\right)\log2/\left(\log\text{N}-\log{\text{N}}_{0}\right) \end{array}$$

where t and t_0_ were the times at which the cells were counted, and N and N_0_ were the cell numbers at times t and t_0_, respectively.

### FACs analysis

Flow cytometry analyses were performed on hDPSCs cultured in medium supplemented with C-FBS or NZ-FBS at first passage of culture. Cells were incubated with FITC-conjugated anti-CD90, PerCP-Cy5.5-conjugated anti-CD105, APC-Cy7-conjugated anti-CD45 (all purchased from BD Pharmingen, San Diego, CA), and PE-conjugated anti-CD34 (Miltenyi Biotech, Calderara di Reno, Bologna, Italy) for phenotypic characterization; and anti-Bone sialoprotein (BSP) (Abcam, Cambridge, UK), anti-CFS-conjugated anti-Osteopontin (OPN), PE-conjugated anti-Osteocalcin (OC) (both from R&D Systems, Minneapolis, MN) and PerCP-Cy 5.5-conjugated anti-Nanog (BD Pharmingen, Milan, Italy) to evaluate osteogenic differentiation and mesenchymal stemness. hDPSCs were sorted by CD34 expression. The purity of sorting was 90%. As negative controls, cells were stained with an isotype control antibody. After incubation with the antibody, cells were resuspended in PBS and analyzed with a FACS ARIA III (BD Biosciences, San Jose, CA). For intracellular staining of Osteocalcin, Osteopontin and Nanog, cells were processed using Fix & Perm Kit (Invitrogen, Milan, Italy) following the manufacturer's guidelines. All data were analyzed using FCS express version 3 (De Novo Software, Glendale, CA).

### RNA isolation and qRT-PCR

Total RNA was extracted from hDPSCs after 7, 14, and 21 days of culture in DMEM supplemented with 10% C-FBS or 10% FBS New Zealand, using an AMBION kit (Life Technologies Italia, Monza, Italy) following the manufacturer's instructions. RNA was treated with DNase (Promega, Milan, italy) to exclude DNA contamination and stored at −80°C. cDNA synthesis was carried out from total RNA (1 μg) using VILO SUPERSCRIPT (Invitrogen, Monza, Italia,). Samples were analyzed using real-time quantitative PCR. PCR reactions were performed using StepOne Thermocycler (Applied Biosystems, Monza, Italy) and the amplifications were done using the SYBR Green PCR Master Mix (Applied Biosystems, Monza, Italy). The thermal cycling conditions were: 50°C for 2 min followed by an initial denaturation step at 95°C for 2 min, 40 cycles at 95°C for 30 s, 60°C for 30 s and 72°C for 30 s. Real-time PCR was performed using the primer sequences shown in Table 1. The experiments were carried out in triplicate for each data point. Gene expression was normalized to *GAPDH* considered as internal control.

**Table 1:** **Primers for real time RT-PCR**.

**Gene**	**Primer sequence**	**Ta (°C)**
*GAPDH*	Fw: GGAGTCAACGGATTTGGTCG	57
	Rev: CTTCCCGTTCTCAGCCTTGA	
*CD90*	Fw: CCCAGTGAAGATGCAGGTTT	60
	Rev: GACAGCCTGAGAGGGTCTTG	
*RUNX2*	Fw: CACTCACTACCACACCTACC	52
	Rev: TTCCATCAGCGTCAACACC	
*CD34*	Fw: TCAAATGTTCAGGCATCAGAG	56
	Rev: TCAGGTCAGATTGGTGCTT	
*NANOG*	Fw: TTCAGTCTGGACACTGGCTG	58
	Rev: CTCGGTGATTAGGGTCCAAC	
*BGLAP (OSTEOCALCIN)*	Fw: CTCACACTCCTCGCCCTATTG	57
	Rev: CTTGGACACAAAGGCTGCAC	
*SPP1 (OSTEOPONTIN)*	Fw: GCCGAGGTGATAGTGTGGTT	58
	Rev: TGAGGTGATGTCCTCGTCTG	
*IBSP*	Fw: GGGCAGTAGTGACTCATCCG	58
	Rev: TTCTCAGCCTCAGAGTCTTCA	
*SP7 (OSTERIX)*	Fw: TCCTCCCTGCTTGAGGAGGA	60
	Rev: AGTCCCGCAGAGGGCTAGAG	
*CXCR4*	Fw: CCTATGCAAGGCAGTCCATGT	57
	Rev: GGTAGCGGTCCAGACTGATGA	
*ITGB1 (INTEGRIN-BETA1)*	Fw: CATCTGCGAGTGTGGTGTCT	57
	Rev: GGGGTAATTTGTCCCGACTT	
*VEGF*	Fw: TGACAGGGAAGAGGAGGAGA	59
	Rev: CGTCTGACCTGGGGTAGAGA	
*PDGFA*	Fw: ACACGAGCAGTGTCAAGTGC	60
	Rev: GGCTCATCCTCACCTCACAT	

### Alizarin red staining and quantification

After 21 days of culture in the different sera, osteogenic differentiation was evaluated by Alizarin Red staining to visualize calcium-rich deposits produced by the cells. The samples were washed twice in PBS, fixed with 4% paraformaldehyde (PFA) in PBS for 30 min at 4°C, and stained with Alizarin Red solution (2%, pH 4.2; Sigma Aldrich, Milan, Italy) for 20 min at room temperature. Stained cells were extensively washed with deionized water to remove any nonspecific precipitation. Micrographs were taken with a microscope Eclipse TE2000-S (Nikon, Firenze, Italy) and a Nikon camera (Nikon, Firenze, Italy).

For the quantification of Alizarin Red S staining, 4 ml 10% (vol/vol) acetic acid was added to each well and the plate was incubated at room temperature for 30 min under gentle agitation. The monolayer was scraped off the plate and transferred to a 1.5 ml microcentrifuge tube after adding 1 ml of 10% (vol/vol) acetic acid. After vortexing for 30 s, the slurry was overlaid with 1.25 ml mineral oil (Sigma-Aldrich, St. Louis, MO), heated to 85°C for 10 min, and transferred to ice for 5 min. The slurry was then centrifuged at 20,000 g for 15 min, and 500 μl of the supernatant then removed to a new 1.5 ml microcentrifuge tube. 200 μl of 10% (vol/vol) ammonium hydroxide was added to neutralize the acid. Absorbance of aliquots (150 μl) of the supernatant was measured in triplicate at 405 nm in a 96-well format.

### Quantification of osteocalcin in supernatant

Osteocalcin levels were evaluated in the culture supernatants of hDPSCs at different time and tested sera by a solid phase Enzyme Amplified Sensitivity Immunoassay (EASIA) kit (Invitrogen, Monza, Italy). Standards and samples were analyzed in duplicate. The assay was performed according to the manufacturer's protocols. The absorbance of each well was read at 450 nm and the concentration of Osteocalcin was determined by interpolation from the standard curve.

### Cell seeding on bio-gide® collagen scaffolds

In order to both better analyze the hDPSCs performances in presence of the two sera, and understand the DPSCs's potential differentiation capabilities, we challenged these stem cells in a 3D culture system, in order to determine to what extent these cells were capable of differentiating toward bone and vessel progenitors, in presence of GMP-approved or FBS serum. For this purpose, hDPSCs were seeded onto BioGide® (Geistlich Pharma AG, Switzerland), a clinical-grade collagen I reabsorbable bilayer membrane composed of a superior thick and an inferior smooth surface. The scaffold's membrane is made of a natural collagen I without further cross-linking: the superior thick and porous surface allows the adhesion of osteoblasts on the surface, whereas the dense and smooth surface hinders cells to both adhere and penetrate inside it.

This scaffold, commercially used in dentistry, has a low antigenicity and excellent biocompatibility.

The membranes were cut under sterile conditions into pieces of equal size (5 × 5 mm). Scaffolds were placed in 6-well-plates and a cell suspension of 1 × 10^6^ cells/ 200 μl of medium was pipetted onto the top (thick and porous surface) of each piece. Cells were allowed to adhere under a humidified atmosphere at 37°C and 5% CO_2_ for 4 h. The seeded scaffolds were then placed in tubes containing DMEM supplemented with the two different sera (C-FBS and NZ-FBS) for 30 days in an incubator at 37°C and 5% CO_2_. Media were changed twice a week.

### Cryostat sectioning

After 3D culture, samples were fixed in 4% paraformaldehyde (PFA) and cryoprotected overnight at 4°C by immersion in a 30% (wt/vol) sucrose solution before being embedded in Tissue-Tek® O.C.T. Compound (Tissue-Tek; Sakura Finetek, Torrance, CA,) and frozen. Sections were cut 5-μm thick with a cryostat at −20°C and then processed for immunostaining.

### Immunohistochemistry/immunofluorescence analysis

hDPSCs cultured in 24-well plates were fixed with 4% PFA in PBS for 20 min at room temperature. After washing in PBS, samples were permeabilized with 0.1% Triton X-100 for 5 min and blocked with 1% BSA in PBS. Incubation with primary anti-OC antibody (sc-30044 Santa Cruz) was performed overnight at 4°C. Primary antibody was revealed using a FITC-conjugated anti-rabbit IgG secondary antibody. Nuclei were stained with Hoechst.

Frozen sections were stained by Hematoxylin and Eosin or permeabilized with 0.1% Triton X-100 for 15 min. Incubation with primary antibodies anti-OPN (ab8448 Abcam, Cambridge, UK), anti-OC (sc-30044 Santa Cruz), and anti-VEGF (ab39250 Abcam Cambridge, UK) was performed overnight at 4°C. FITC conjugated secondary antibody to rabbit IgG was used to reveal primary antibodies. Micrographs were taken with a microscope EVOS FL Cell Imaging System (Life Technologies).

### Statistical analysis

Values are shown as the mean ± S.E.M. of measurements of at least three independently performed experiments to avoid possible variation of cell cultures. Student's *t*-test was employed, and *p* < 0.05 was considered to be statistically significant.

## Results

### Characterization of hDPSCs by flow cytometric analysis

**Figure 1:**
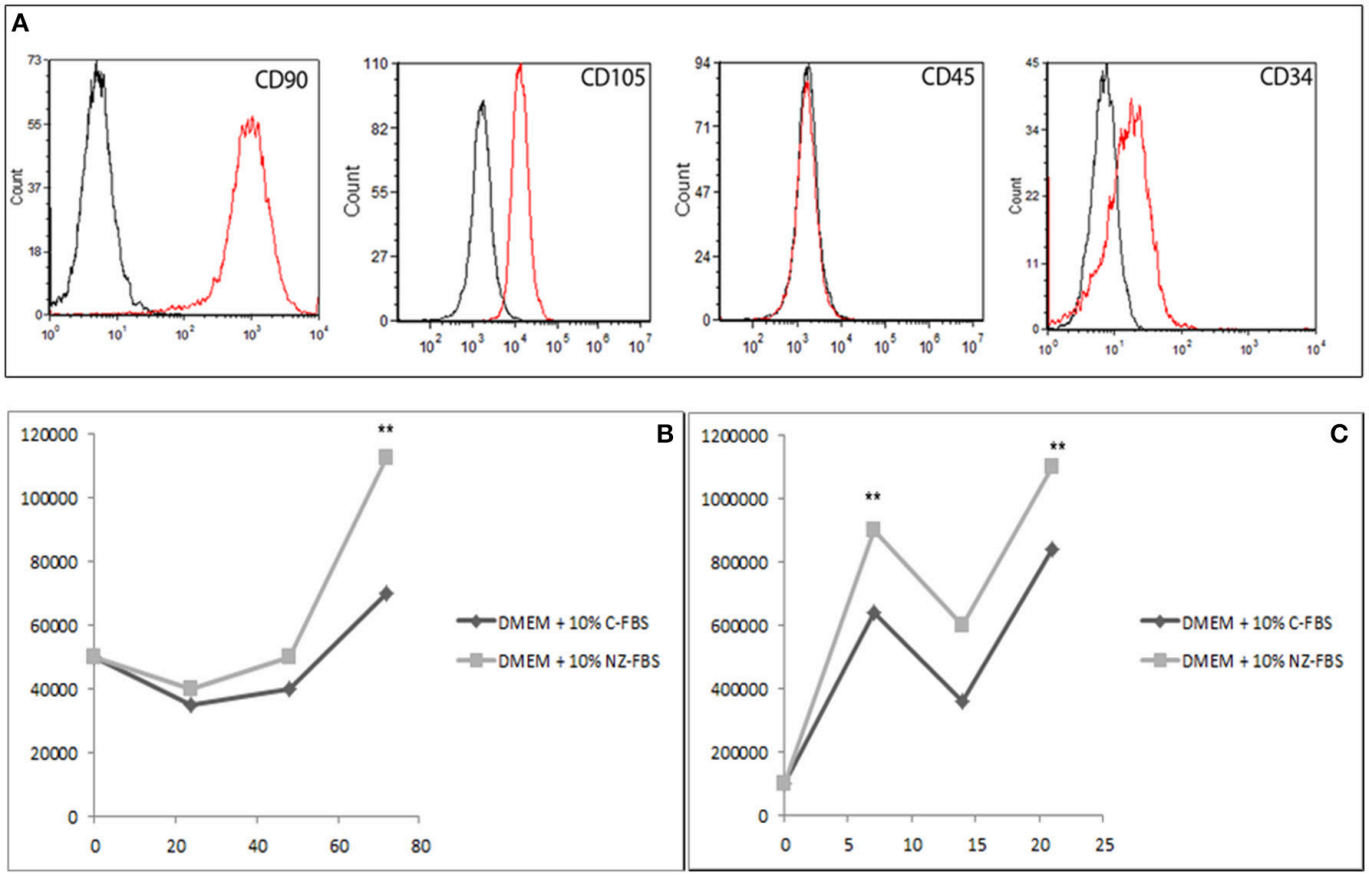
**(A)** Characterization of DPSCs at the first passage of culture: cytometric analysis of CD90, CD105, CD45, and CD34 markers in DPSCs. Histograms represent the number of cells (y axis) and the fluorescence intensity (x axis) relative to unstained control cells (dot line) and cells marked with specific antibodies against surface proteins (solid line). **(B)** Growth curve of DPSCs cultured in DMEM + 10% of standard FBS and in DMEM + 10% of New Zealand FBS up to 72 h. **(C)** Growth performance was studied up to 21 days. ^**^p < 0.001.

At the first culture passage, cells were characterized by evaluating the expression of CD90, CD105, CD45, and CD34 using cytometric analysis. Cells were all positive for the mesenchymal markers CD90 and CD105, and negative for CD45. Approximately 20% of cells were positive for CD34 (Figure 1A). Then cells were sorted by CD34.

### Evaluation of cell proliferation after sera treatment

Cell proliferation under the different conditions was evaluated at 24, 48, and 72 h (Figure 1B) and at 7, 14, and 21 days (Figure 1C) of culture. To this purpose, equal numbers of cells were initially plated and the relative time of duplication was assessed. Either C-FBS and NZ-FBS promoted hDPSC proliferation. However, there were lower numbers of cells in bovine-origin sera compared with NZ-FBS, a difference already detectable at 48 h (Figure 1B). NZ-FBS stimulated earlier cell duplication with a reduction in the doubling time to less than a half of that of NZ-FBS (Table 2).

**Table 2:** **Doubling time of DPSCs under different culture conditions**.

**Culture conditions**	**Doubling Time (hours)**	***P*-value**
DMEM + 10% C-FBS	126 ± 12	*P* < 0.001
DMEM + 10% NZ-FBS	94 ± 7	*P* < 0.005

### Phenotypic characterization of DPSCs during cell culture

**Figure 2:**
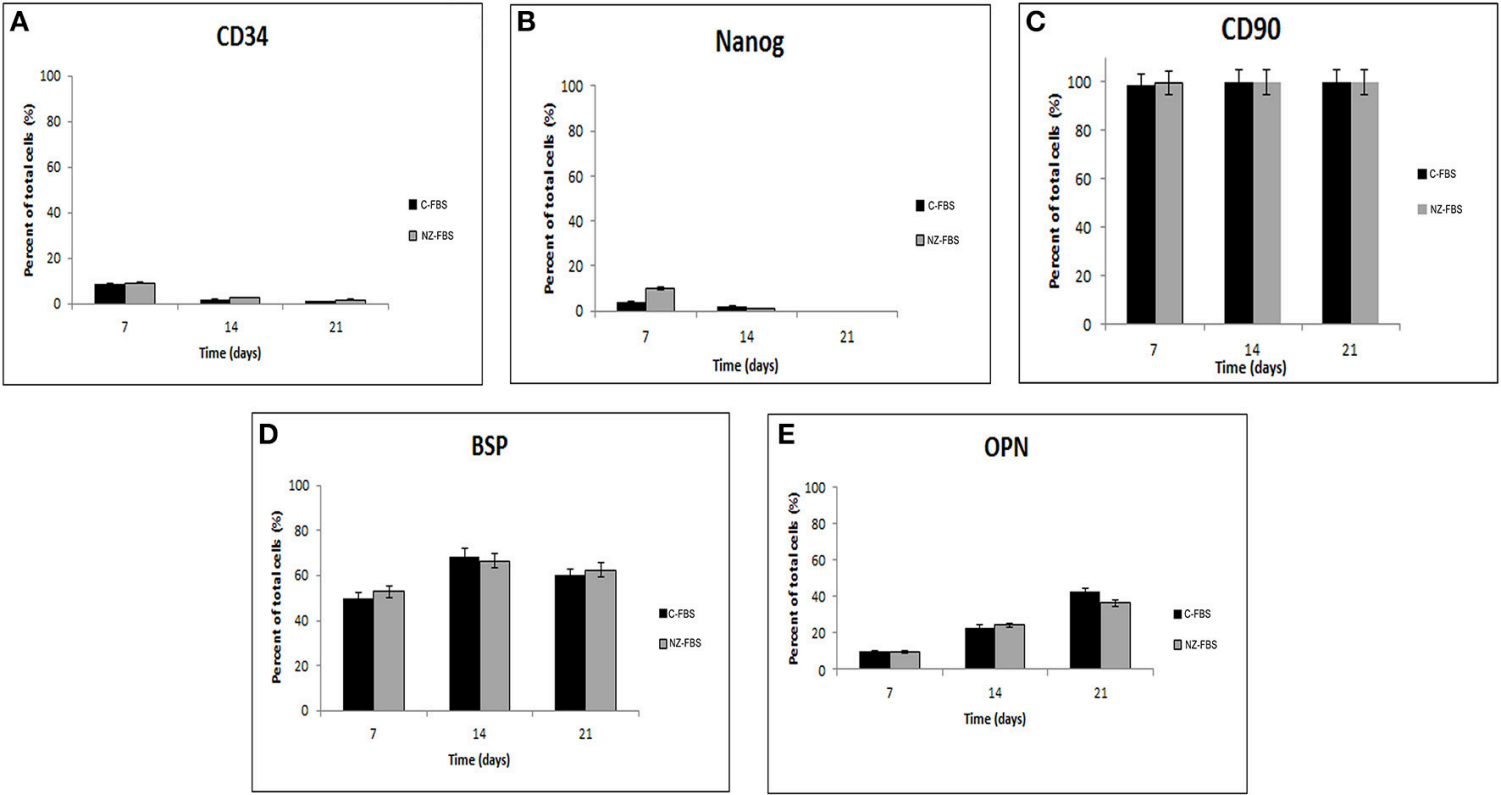
**Cytometric analysis for stemness markers (A–C), and osteogenic markers (D,E) in DPSCs**.

In order to evaluate the effect of different sera on the expression of stemness and osteogenic markers, a flow cytometric assay at different days of culture for CD34, NANOG, CD90, BSP, and OPN, was performed (Figures 2A–E). Levels of the stromal-vascular antigen CD34 decreased up to day 21 (Figure 2A) in both sera. Expression of the stemness marker NANOG also decreased, although its protein level was higher at 7 days of culture in NZ-FBS (Figure 2B). All hDPSCs expanded with C-FBS and NZ-FBS, showed positivity (>98%) for the mesenchymal marker CD90 (Figure 2C).

The expression of osteogenic markers BSP and OPN did not show substantial differences during culture time using both sera (Figures 2D,E).

**Figure 3:**
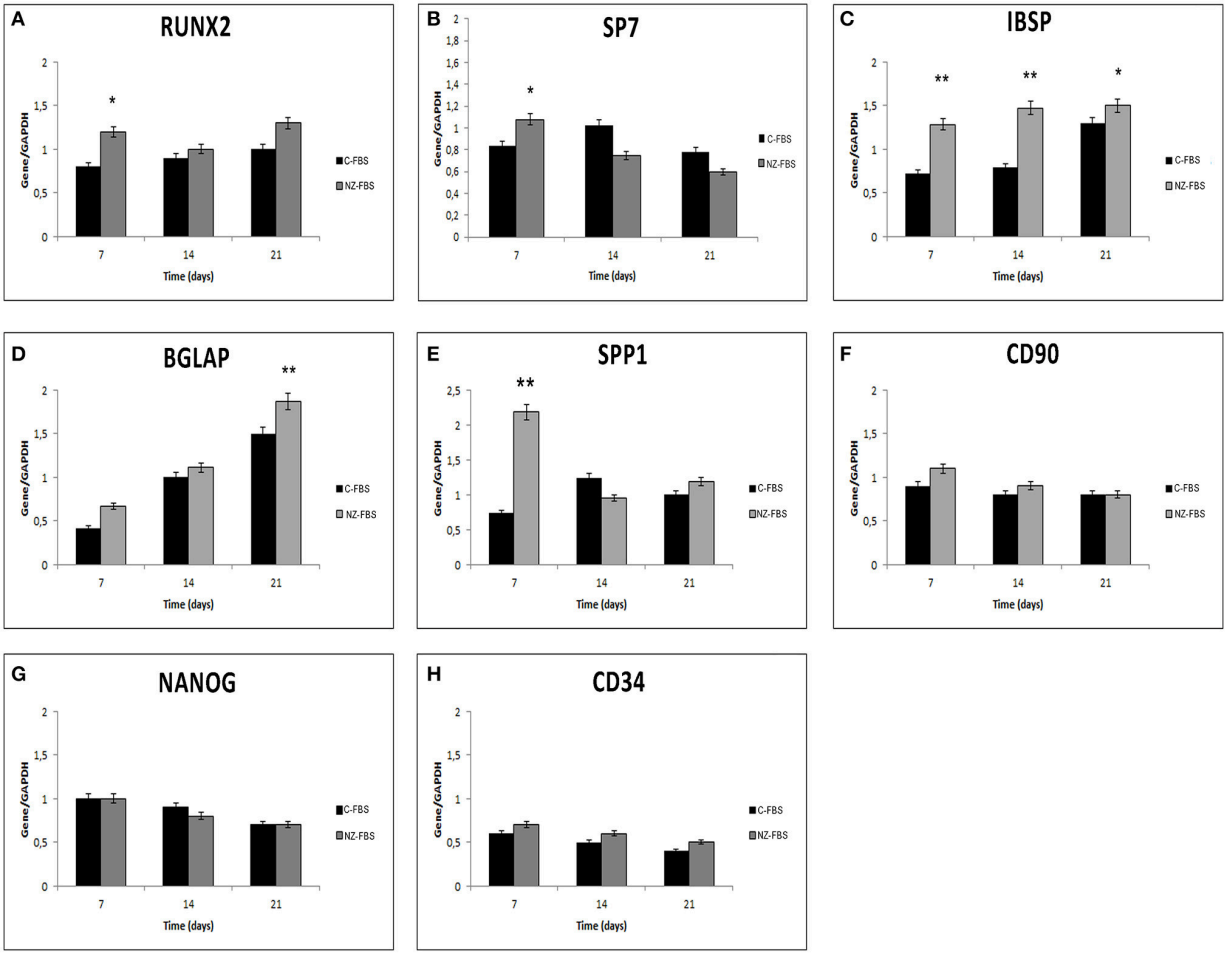
Gene expression analysis for osteogenic markers (A–E), and stemness markers (F–H) in DPSCs. ^*^p < 0.05; ^**^p < 0.001.

Further investigations on osteogenic features and the maintenance of stemness were also performed using a quantitative RT-PCR for *RUNX2, SP7 (OSTERIX), IBSP, BGLAP(OSTEOCALCIN), SPP1 (OSTEOPONTIN), CD90, NANOG*, and *CD34* at 7, 14, and 21 days of culture (Figures 3A–H). The expression of *RUNX2*, the master transcription factor of osteogenic differentiation, was higher in FBS-NZ at 7 days of culture and then decreased at 14 and 21 days in both sera without significant differences (Figure 3A). *SP7* mRNA, another transcription factor involved in osteogenic differentiation, showed an increase only at 7 days of culture (Figure 3B).

mRNA levels of *IBSP, BGLAP* and *SPP1* increased in cells cultured with FBS-NZ starting from 7 days (Figures 3C–E).

The mRNA levels of *CD90* were comparable in all the experimental conditions (Figure 3F). *NANOG* was always expressed but decreased during culture time as well as *CD34* mRNA (Figures 3G,H).

### Intracellular localization of osteocalcin, evaluation of mineralization, and osteocalcin secretion

**Figure 4:**
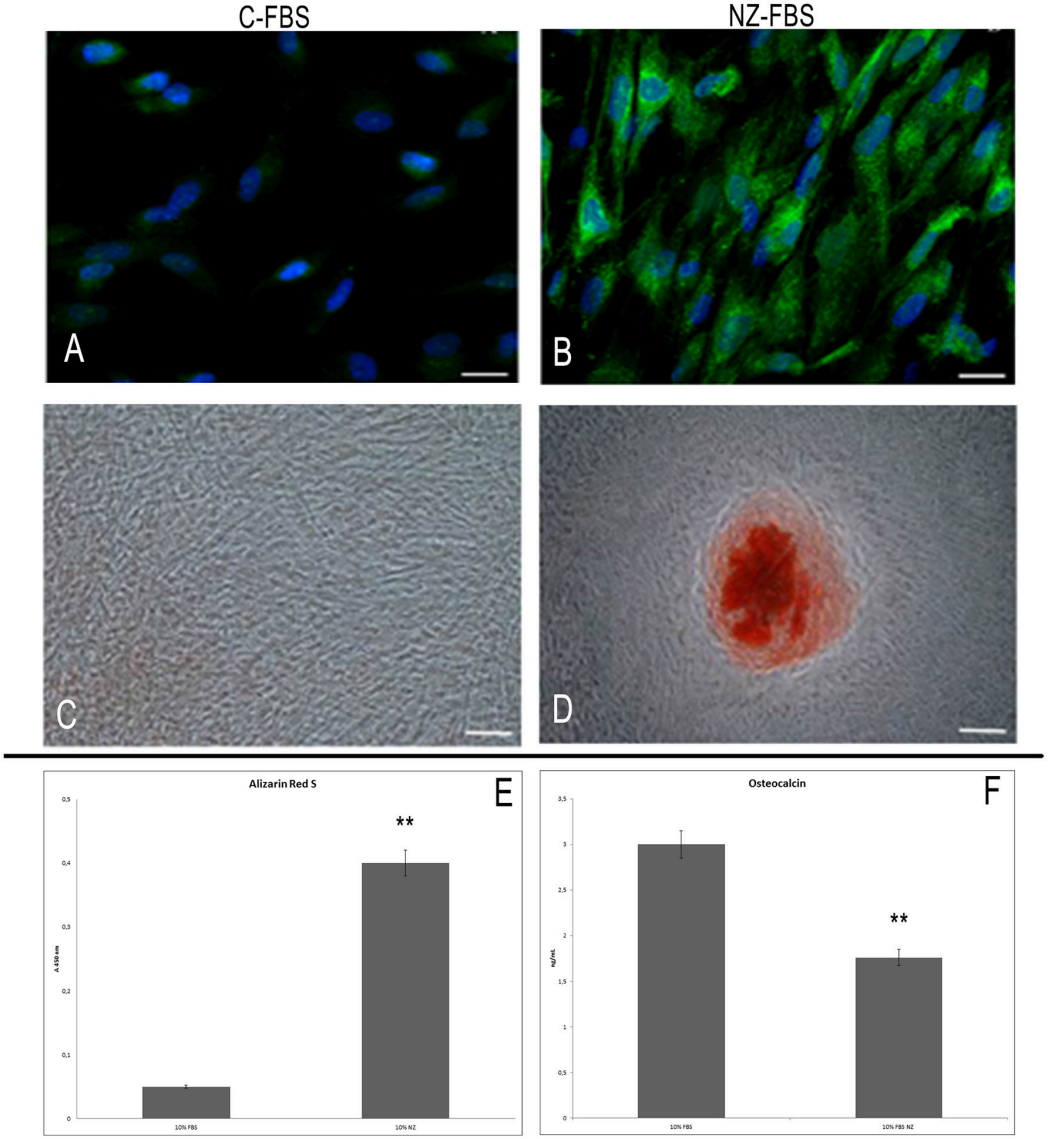
**Immunofluorescence analysis for osteocalcin in DPSCs cultured with C-FBS (A) and NZ-FBS (B)**. Scale bar = 10 μm. Alizarin red analysis of DPSCs cultured with C-FBS **(C)** and NZ-FBS **(D)**. Scale bar = 100 μm. **(E)** Alizarin Red Quantification. **(F)** Osteocalcin concentration with C-FBS and NZ-FBS. ^**^p < 0.001.

Immunoreactivity for OC was strongest and distributed throughout the cell in hDPSCs cultured with NZ-FBS at 21 days of culture (Figure 4B). hDPSCs cultured with C-FBS were only weakly positive for OC that was mostly localized in the perinuclear region (Figure 4A).

Alizarin red staining was used to evaluate calcium-rich deposits after 21 days of culture (Figures 4C,D). Calcified nodules were absent in cultures with C-FBS (Figure 4C) but small areas of staining were evident, whereas mineral deposition was observed in cultures with NZ-FBS (Figure 4D). This observation was confirmed by Alizarin red quantification (Figure 4E).

Moreover, we determined the concentration of OC in the culture supernatants after 21 days. A significant difference was observed between the two tested sera (Figure 4F). In FBS-NZ the amount of osteocalcin in the medium was very low.

### Homing, chemotaxis and evaluation of angiogenesis

**Figure 5:**
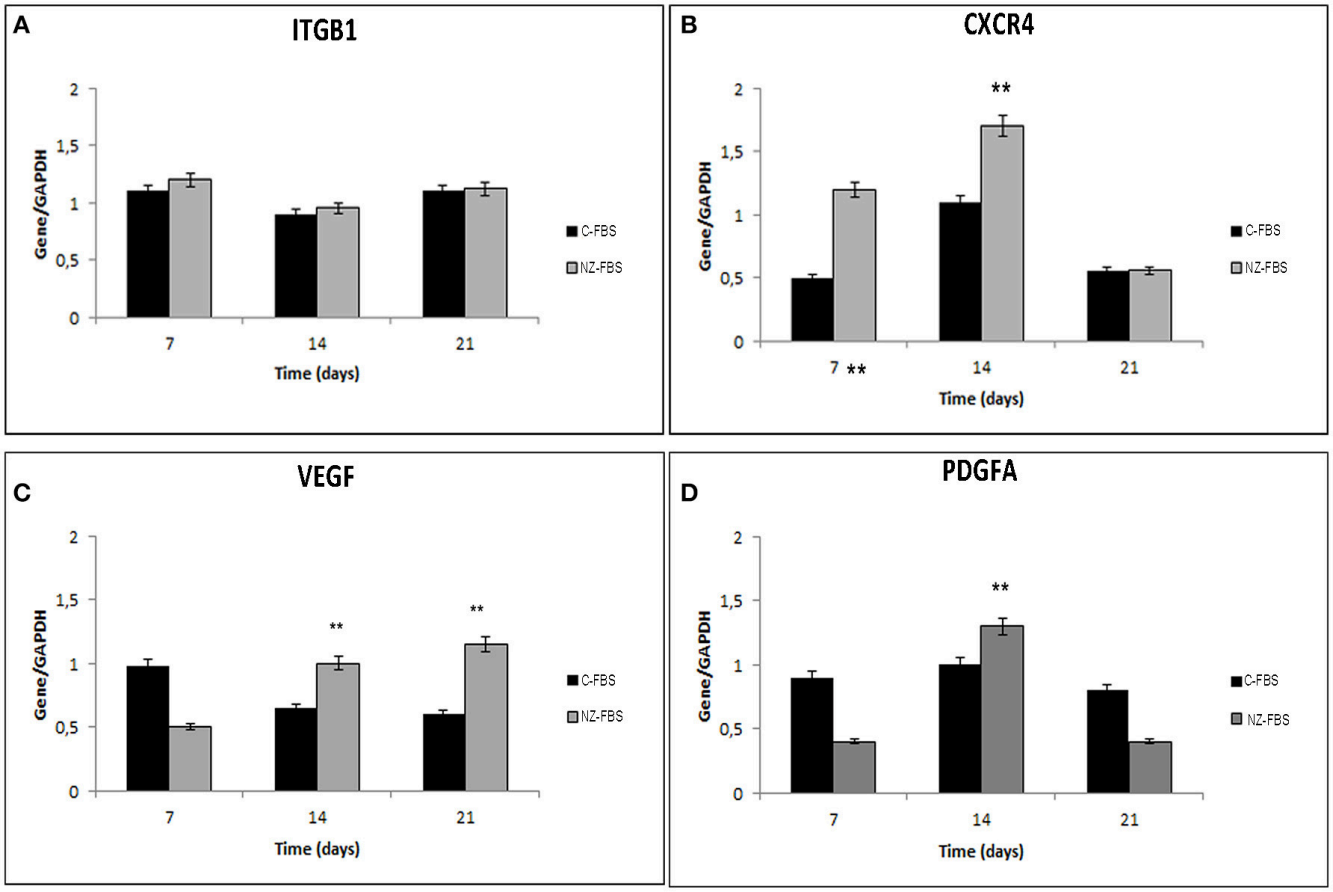
**Real time PCR for genes involved in chemotaxis and cell adhesion**. In **(A,B)** are respectively shown expression of Integrin beta 1 (Intβ1) and chemokine receptor 4 (CXCR4) mRNA in DPSCs. Real time PCR for genes involved in angiogenesis: VEGF **(C)** and PDGFA **(D)** mRNA levels in DPSCs. All results were normalized to GAPDH expression. ^**^p < 0.001.

In order to evaluate a possible effect of FBS-NZ on DPSCs homing and chemotaxis, we assessed the expression of *ITGB1* (*INTEGRIN*-β*1*) and *CXCR4. ITGB1* mRNA showed a slight increase at 7 days of culture with NZ-FBS, but then remained almost unchanged in the two different tested sera (Figure 5A). The expression of *CXCR4* was more induced by NZ-FBS at 7 and 14 days of culture, and drastically decreased at 21 days (Figure 5B).

The expression of *VEGF* and Platelet-derived growth factor A (*PDGFA*) mRNAs in hDPSCs was also evaluated after numerous days of culture (Figures 5C,D). The *VEGF* expression was significantly promoted by NZ-FBS at 14 and 21 days of culture (Figure 5C); the expression of *PDGFA* gene was higher in NZ-FBS samples only at 14 days of culture, then decreased (Figure 5D). hDPSCs were negative for *PDGFB* mRNA in all the experimental conditions (data not shown).

### 3D culture experiments

**Figure 6:**
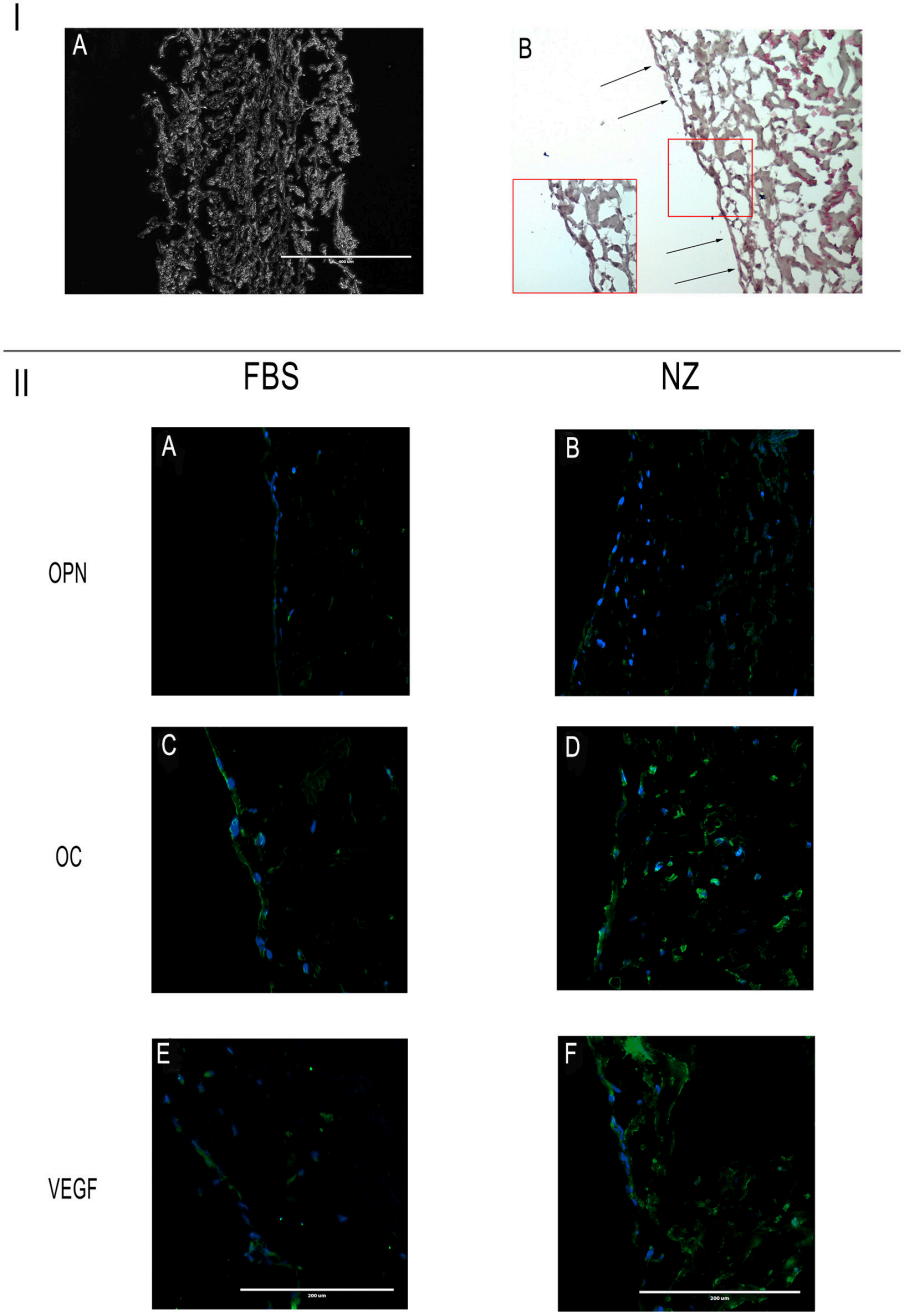
**I: (A)** Cross section of the scaffold; **(B)** hDPSCs on the scaffold's surface constitute a cell monolayer. Scale bar = 400 μm. **II:** Immunofluorescence analysis of specific antibodies for osteo-angiogenesis, (OPN, OC, VEGF) in cells seeded on scaffolds and cultured with C-FBS **(A,C,E)** and NZ-FBS **(B,D,F)**. Scale bar = 200 μm.

The results of 3D cultures are shown in Figure 6. In this figure, the cross section of the scaffold is depicted in IA: it is made of collagen I fibers surrounded on the two sides by a membrane (one thick and porous and another smooth).

Challenging hDPSCs with this scaffold, in presence of the two sera, hDPSCs displayed on the scaffold's surface constituting a cell monolayer as shown in Figure 6IB. Specifically, through the use of specific antibodies for osteo-angiogenesis, we found that cells expressed all the markers, including OPN, OC, VEGF (Figures 6IIA–F), through the whole cell layer.

In particular, using NZ-FBS in cultures, DPSCs were found to be noticeably positive for the said markers of bone and vessels (Figures 6IIB,D,F).

## Discussion

The use of animal sera for stem cell cultures may be unsafe for human cell-based therapies as they may contain factors or pathogens that could be transferred to patients or cause immune rejection during cell transplantations (Mackensen et al., 2000; Sundin et al., 2007).

In the present study, a GMP-approved serum was compared with a commercial fetal bovine serum during isolation and expansion of hDPSCs *in vitro*.

Freshly isolated hDPSCs, were characterized and found to be positive for common mesenchymal stem cells markers. Later, hDPSCs phenotype was characterized during culture and the differences in cell proliferation, stemness, and osteogenic features were assessed.

In this study it has been shown that NZ-FBS induced significant cell growth with respect to C-FBS. The expression of CD90 marker remained unchanged throughout all experimental conditions, indicating that these cells maintained the mesenchymal lineage, whereas the expression of stemness markers CD34 and NANOG decreased with time, as expected. This suggested that DPSCs progressively lose their stem cell property during *in vitro* expansion. In addition, we observed that the use of NZ-FBS promoted an earlier increase expression of osteogenic markers, in particular of those involved in the formation of mineralized matrix (BSP and OPN) within 14 days as well as evidenced by Alizarin Red staining. Moreover, the osteocalcin expression in 2D culture, during time, showed a significant increase: *BGLAP* mRNA was higher with NZ-FBS compared with C-FBS cultures, and its protein detected by immunofluorescence, was more evident and distributed throughout the cytoplasm. In addition, we noted that the lowest amount of osteocalcin was found in medium recovered from cells cultured with NZ-FBS. This suggested that released osteocalcin can bind to the extracellular matrix and guide hydroxyapatite to form mineralized nodules (Neve et al., 2013).

*RUNX2* and *SP7* are the key transcription factors initiating and regulating the osteogenesis, respectively (Zhou et al., 2010; Bruderer et al., 2014). NZ-FBS induced a slight increase in *RUNX2* and *SP7* at 7 days of culture. In summary, NZ-FBS induced a positive effect on DPSCs culture allowing an earlier commitment to differentiation toward osteogenic fates, when compared with C-FBS. The differences in osteogenic potential were detectable already at 7 days of culture, when NZ-FBS enhanced the expression of bone-related markers.

When a tissue or organ is damaged, several mechanisms may contribute to the success of wound repair, both under physiological conditions and after administration of stem cells. Angiogenesis, chemotaxis, homing, and engraftment are crucial in equal measure to allow the repair of an injured tissue by stem cells. Chemotactic stimuli recruit various cell types to the injury site. The interaction between the chemokine SDF1 and its receptor CXCR4 expressed on the hDPSCs' surface is a key factor in the recruitment of these cells to the damaged area (Jiang et al., 2008; Kim et al., 2013). The use of both sera induced an increase in *CXCR4* expression on hDPSCs within 14 days of culture but this was more evident in cells expanded with NZ-FBS. In addition to cell migration, cell-matrix, and cell-cell interactions play essential roles in tissue reparation. The expression of surface molecules such as integrins may influence the maintenance of stemness, cell proliferation and changes during the differentiation process. Our findings show that there are no substantial differences in the effects of integrins between the various sera, suggesting that culture conditions did not interfere with cell adhesion.

When an injury, such as a fracture occurs, the vasculature is compromised and hinders the supply of nutrients and oxygen to the site of the lesion. Angiogenesis is a crucial process for bone healing. During tissue reparation, the migration and organization of endothelial cells into capillary tubes is regulated by a paracrine and autocrine action of VEGF (Lamalice et al., 2007). When the damaged tissue involves bone, the cells located in its inner part have a limited blood and oxygen supply. This major issue must necessarily be overtaken in order to obtain good results in clinical practice. At this purpose, we considered the expression of *VEGF* and *PDGFA*. hDPSCs cultured in presence of NZ-FBS were found to produce higher mRNA levels of the said angiogenic factors.

In addition, hDPSC's capabilities in culture were examined by evaluating a 3D environment. To this aim, we seeded hDPSCs on the Bio-Gide scaffold.

This scaffold was chosen because of the following features: it is made of collagen I, the physiological collagen of bone; it is clinically approved and commonly used mainly in order to constitute a biological barrier between bone and soft tissues. In fact, during surgical procedures such as craniofacial and orthopedic surgeries, the correct growth of both bone and the surrounding connective tissue must be ensured. For this purpose, the aforementioned scaffold has been manufactured as a biological barrier to cell penetration on one of the two surfaces: one thick and porous (upper) and another smooth (lower), generally facing the soft tissues. The thick surface allows the ingrowth of bone-forming cells, whereas the latter prevents the ingrowth of fibrous tissue into the bone defect. Therefore, cells are not allowed to penetrate it and they cannot invade the re-absorbable scaffold. Thereby, the cells remain on the surface and our aim, devoted to observe the level of differentiation under the different sera, was better evidenced by observing them at the surface of the scaffold, without other possible interferences. Finally, based on the clinical observations, this scaffold is fully biocompatible. As evidenced by the results, hDPSCs on the scaffold's surface strongly expressed OC, OPN and VEGF in all the cases above all in presence of NZ-FBS. Taken together, our results strongly indicate that NZ-FBS (clinical grade) has a positive effect on replicative potentials and self-commitment of hDPSCs. Therefore, NZ-FBS can be considered a reliable tool for cell-based treatments in autologous grafts and GMP conditions for bone repair and regeneration. Under these conditions, hDPSCs retain their features directing toward osteo-angiogenesis lineage, which represents the gold standard in order to obtain a well-vascularized bone.

In addition, the use of sera belonging to controlled animals could overcome the problems related to GMP standards, that require developing well-defined culture conditions, guaranteeing the maintenance of stem cell multipotency and/or differentiation.

## Author contributions

MN: Designed the study, performed the experiments, wrote the manuscript and approved it. AS, RM, DL, LL, AR: Performed experiments, collected data. TM: Designed the experiment, approved the manuscript.

### Conflict of interest statement

The authors declare that the research was conducted in the absence of any commercial or financial relationships that could be construed as a potential conflict of interest. The handling Editor declared a collaboration with one of the authors TM and states that the process nevertheless met the standards of a fair and objective review.
